# Community ecological succession of endophytic fungi associates with medicinal compound accumulation in *Sophora alopecuroides*

**DOI:** 10.1128/spectrum.03076-23

**Published:** 2024-01-18

**Authors:** Mingxiu Ju, Qingchen Zhang, Ruotong Wang, Siyuan Yan, Qiangqiang Zhang, Peng Li, Fengxia Hao, Peiwen Gu

**Affiliations:** 1 College of Forestry and Prataculture, Ningxia University, Yinchuan, China; 2 Department of Pharmacotherapy and Translational Research, University of Florida, Gainesville, Florida, USA; 3 School of Agriculture, Ningxia University, Yinchuan, China; 4 State Key Laboratory of High-Efficiency Utilization of Coal and Green Chemical Engineering, Ningxia University, Yinchuan, China; Agroscope, Nyon, Switzerland

**Keywords:** *Sophora alopecuroides*, community characteristics, core microbiome, quinolizidine alkaloids, co-occurrence network analysis

## Abstract

Endophytic fungi of medicinal plants are symbiotic with the host and play an important role in determining metabolites. To understand the relationship between the accumulation of *Sophora alopecuroides*’ medicinal bioactive compounds and the ecological succession of endophytic fungi, here we collected samples from *S. alopecuroides* at four developmental stages (adult, flowering, podding, and mature) and different organs (roots, stems, leaves, and seeds) at the mature stage. We then used high-performance liquid chromatography-mass spectrometry and high-throughput sequencing on the internal transcribed spacer region to identify the medicinal compounds and endophytic fungal communities in each sample. The endophytic fungal community characteristics and accumulation of medicinally bioactive compounds of *S. alopecuroides* varied with the host’s developmental stages and organs, with the highest total alkaloids content of 111.9 mg/g at the mature stage. Membership analysis and network connection analysis showed a total of 15 core endophytic fungi in different developmental stages and 16 core endophytic fungi in different organs at the mature stage. The unclassified Ascomycota, *Aspergillus*, and *Alternaria* were significantly and positively correlated with the medicinal compounds of *S. alopecuroides* at the mature stage (*r* > 0.6 or *r* < −0.6; *P* < 0.05). In this study, we identified key endophytic fungal resources that affect the content of medicinally bioactive compounds in *S. alopecuroides*. This discovery could lay the foundation for enhancing the yield of medicinally bioactive compounds in *S. alopecuroides* and the development and application of functional endophytic fungi.

**IMPORTANCE:**

*Sophora alopecuroides* is a traditional Chinese herbal medicine. The major medicinal chemicals are considered to be quinolizidine alkaloids. Quinolizidine alkaloids have been widely used for the treatment of tumors, dysentery, and enteritis. Previous studies have found that endophytic fungi in *S. alopecuroides* can promote the accumulation of host quinolizidine alkaloids. However, the relationship between the accumulation of *S. alopecuroides*’ medicinal bioactive compounds and the ecological succession of endophytic fungi remains unclear. In this study, we screened the key endophytic fungal resources affecting the content of medicinally bioactive compounds and laid the foundation for subsequent research on the mechanism by which endophytic fungi promote the accumulation of medicinally bioactive compounds in *S. alopecuroides*.

## INTRODUCTION

*Sophora alopecuroides* L. (Kudouzi in Chinese) is a perennial herb in the Fabaceae family. It is mainly distributed in arid and semi-arid regions of Asia. In China, it is found in the northwestern provinces of Xinjiang, Inner Mongolia, Qinghai, Gansu, and Ningxia (1, 2). *S. alopecuroides* has a well-developed root system that allows it to tolerate drought, salinity, and barrenness (1, 3). It is also a pioneer vegetation for environmental protection in desert grasslands, as it is suitable for windbreak and sand fixation (3). *S. alopecuroides* has been used as a traditional medicine in China for centuries. It is clinically used for anti-inflammatory and pain-relieving effects (1, 4). The primary medicinally bioactive compounds in *S. alopecuroides* are quinolizidine alkaloids (QAs), including oxymatrine (OMA), matrine (MA), sophridine (SR), sophocarpine (SC), and oxysophocarpine (OSC) (5–7). Artificial cultivation of *S. alopecuroides* is difficult and uneconomical, due to low germination rates, slow growth, high costs, and most importantly, low yield of QAs (8). This makes it difficult to meet the large demand in the medicinal market (8, 9). Additionally, the harvesting of *S. alopecuroides* can potentially cause ecological problems, such as the degradation of grasslands and the destruction of the ecological balance in associated regions (3). Therefore, there is an urgent need to develop new methods to increase the content of medicinal compounds and improve the medicinal quality of *S. alopecuroides*.

The abundance of bioactive compounds in medicinal plants is closely related to their therapeutic efficacy and is an important indicator of the quality of botanical products (10, 11). The accumulation of these compounds is influenced by both internal and external factors (12, 13). The external factors include elevation, temperature, humidity, soil fertility, and pH, while the internal factors include the plant species, age, and phenological period (13). The distribution and productivity of medicinal compounds also vary at different maturity stages and in different organs and tissues of the plant (10, 14). For example, during the whole growth period from the pre-blossom, blossom, fruit, and fruit mature periods until the pre-withering period, it was in the fruit mature period that both the total saikosaponin content and the saikosaponin-a content reached the highest level (15). A large number of studies have shown that endophytic fungi are one of the most important internal factors regulating medicinal compounds (16). However, there are many unanswered questions about the dynamic and mechanism of regulation of medicinal compounds by endophytic fungi in medicinal plants at different developmental stages and in different organs, and this topic has received a lot of attention in the area.

Endophytic fungi can directly or indirectly regulate the synthesis and accumulation of secondary metabolites in the host plant, which can affect the distribution and content of bioactive substances in medicinal plants (10, 17). Endophytic fungi regulate the secondary metabolism of medicinal plants mainly in the following ways: (i) endophytic fungi convert and transport nutrients from the rhizosphere to the host or produce growth hormones to ensure plant growth and metabolic material bases (18, 19). (ii) Endophytic fungi infect the plant body, stimulate or induce a host defense response, and trigger the production of secondary metabolites (20, 21). (iii) Endophytic fungi produce the same or similar metabolic compounds as the host and participate in the biotransformation of the host metabolites (17, 22). (iv) Interactions between endophytic fungal communities and between endophytic fungi and their hosts maintain a delicate dynamic balance and chemical exchange, thus affecting the metabolic composition of plants (10). Previous studies by our research group have shown that *S. alopecuroides* is a host of a large number of diverse endophytic fungi (2, 23). The most common and abundant endophytic fungi in *S. alopecuroides* are unclassified Ascomycota, *Tricholoma*, *Apiotrichum*, *Alternaria*, and *Aspergillus* (2, 23). The dominant genera, endemic genera, and biomarkers of endophytic fungi in the four organs (roots, stems, leaves, and seeds) of *S. alopecuroides* were different, indicating that the distribution of endophytic fungi in *S. alopecuroides* is organ specific (23). In northwest China, the diversity of endophytic fungi communities in *S. alopecuroides* is significantly different across geographical regions (2). Preliminary studies have suggested that the abundance of *Alternaria* endophytic fungi is closely related to the synthesis and accumulation of bioactive compounds in *S. alopecuroides* (2). However, the relationship between the ecological succession of endophytic fungi communities in *S. alopecuroides* and their content of medicinal compounds is not yet clear. Further research is needed to better understand the relationship between endophytic fungi communities and the content of medicinal compounds in *S. alopecuroides*.

Co-occurrence network analysis (CNA) is a more powerful tool than simple diversity analysis, which was more commonly used, for studying host-microbe and microbe-microbe interactions (24, 25). CNA can specifically identify the core microbiomes (i.e., the hub microbes) that influence community structure (26, 27). These core microbiomes alter the community microenvironment or plant metabolism by intense biological interactions with the host or other microbial species (27). In the current study, we used plant tissues from different developmental stages (adult, flowering, podding, and mature stages) and different organs (roots, stems, leaves, and seeds) at the mature stage of *S. alopecuroides* to determine the content of QAs in *S. alopecuroides* with liquid chromatography and mass spectrometry (LC-MS). Furthermore, we analyzed the endophytic fungal community structure of *S. alopecuroides* using high-throughput sequencing technology and CNA to identify the core microbiome and explore the relationship between the core microbiome and the medicinal bioactive compounds. In this study, we screened the key endophytic fungal resources affecting the content of medicinally bioactive compounds and laid the foundation for the subsequent research on the mechanism of endophytic fungi promoting the accumulation of medicinally bioactive compounds of *S. alopecuroides*.

## MATERIALS AND METHODS

### Plant material and experimental design

**Fig 1:**
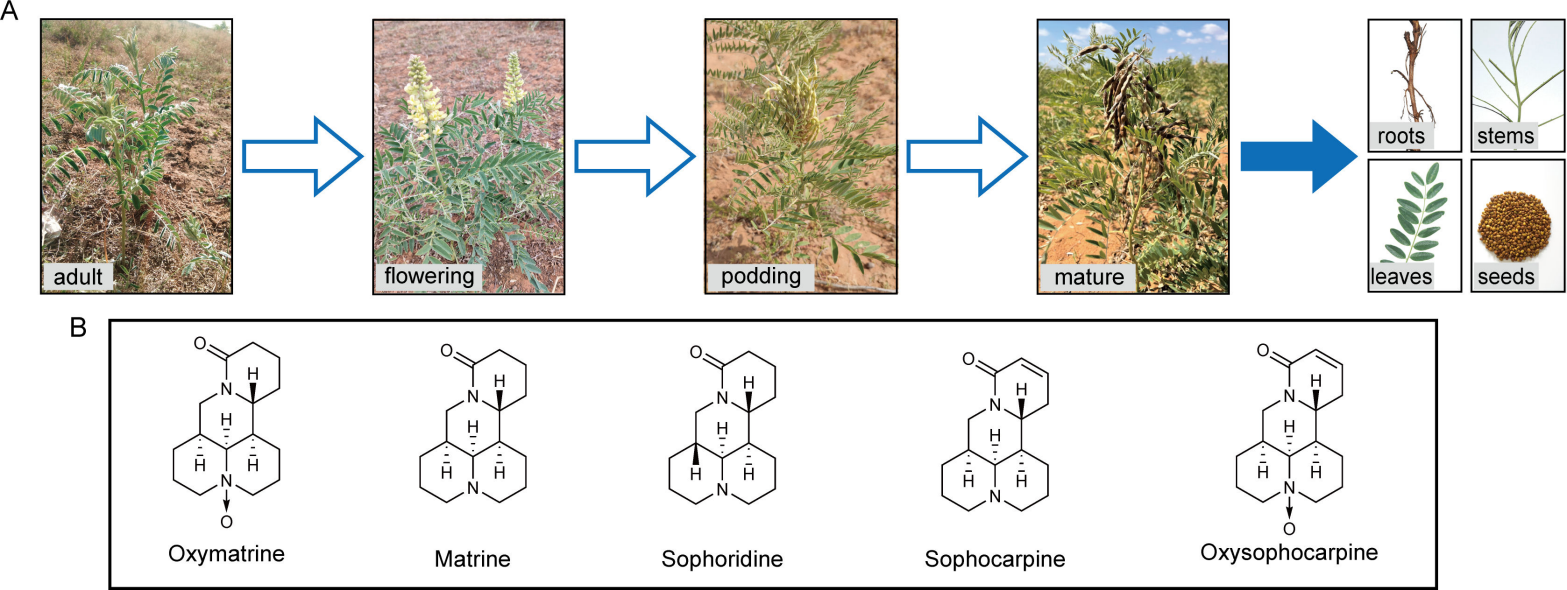
Developmental stages and different organs of *S. alopecuroides* (**A**) and chemical structures of the five quinolizidine alkaloids (**B**).

The test site was located in Huamachi Town, Yanchi County, Ningxia Province (37.77N, 107.46E, altitude at 1,336.4 m), and three sampling points (10 × 10 m) were set up in the meadow where the wild *S. alopecuroides* was the dominant species by diagonal sampling method, with each point spaced more than 150 m apart. *S. alopecuroides* plants were sampled at the adult stage (May), flowering stage (June), podding stage (July), and mature stage (September), respectively (Fig. 1A). After removing 20 cm of dead leaves and debris around the *S. alopecuroides*, we selected healthy, disease-free, pest-free, and mechanically damage-free *S. alopecuroides* plants, excavated the plants about 40 cm vertically downward, and the soil was mulched after sampling for lessening the damage to the ecological environment. A total of 10 plants were collected (sampling distance of 10 m) at each sampling point. The samples were placed in sterile fresh bags, marked and stored at low temperatures, and brought back to the laboratory immediately.

The *S. alopecuroides* plants were separated by roots, stems, leaves, and seed tissues with sterile scissors, and 3 g of each tissue and organ of the same reproductive period were taken and mixed as one sample. There were three replicates for each of the four stages, for a total of 12 samples. In addition, 10 g of roots, stems, leaves, and seeds of *S. alopecuroides* at the mature stage was retained separately for four organs × three replicates, for a total of 12 samples. Both groups of samples were surface disinfected under sterile conditions. The major operations are described as follows: rinsed 3–5 times with sterile water, then sequentially soaked in 75% ethanol for 30 s, with an effective chlorine content of 1% sodium hypochlorite for 1–3 min, then rinsed 3–5 times with sterile water, and then drained with sterile filter paper. To confirm the successful disinfection of the seed surface, the final water rinse was spread on a potato dextrose agar plate and cultured for 3 days in the dark at 25°C. If no colonies were observed, the seeds were assumed to be adequately disinfected and were used for subsequent experiments. Finally, the two groups of samples were divided into two equal parts, one was stored in a sterile tube for high-throughput sequencing of endophyte fungi of *S. alopecuroides,* and the other was pulverized to a homogeneous powder by a freeze-drying grinder (100 mesh) for the extraction and determination of the bioactive compounds. All samples were stored frozen at −80°C in an ultra-low temperature freezer.

### Extraction and content determination of quinolizidine alkaloids from *S. alopecuroides*

The extraction method was modified according to the research of Wang et al. (28). A total of 0.25 g of dried powdered plant samples was placed into a 50 mL conical flask containing 0.08 g cellulase (10 U/g). Next, 40 mL of methanol was added to the conical flask and vortexed to mix for 10 s. The mixture was sonicated at 45°C for 60 min and then centrifuged at 13,000 rpm for 10 min. The supernatant extract was concentrated by removing methanol using a rotary vacuum evaporator at 50°C and dissolved in a 10 mL mobile phase. The solution was passed through a 0.45-µm filter membrane for detection by quantitative and qualitative analysis.

For apparatus and chromatographic conditions, refer to the method of Ju et al. (2). HPLC analysis was performed using Agilent 1260 Infinity II system (Agilent Technologies, Santa Clara, CA, USA). Different bioactive compounds were separated on a reverse-phase column (ZORBAX SBC18 column, 4.6 × 250 mm, 5 µm, Agilent, USA). Chromatographic conditions: mobile phase 0.2% phosphoric acid (triethylamine 2.00 mol/L) (A) and acetonitrile (B), elution gradient 0–22 min, 96.5% A; 22–23 min, 96.5–0% A; 23–27 min, 100% A; 27–28 min, 0–96.5% A; 28–34 min, 96.5% A; detection wavelength: 205 nm; flow rate: 1.0 mL/min; injection volume: 10 µL; and column temperature: 30°C.

#### Preparation of reference standard solution

Each reference standard was prepared by adding a total of 10 mg of either OMA, MA, OSC, or SC reference standard to a volume of 1,000 µL of the mobile phase. Each sample was then semi-sequentially diluted to appropriate concentrations with mobile phase, assayed under the chromatographic conditions described above and a standard curve was drawn (Table S1). Qualitative analysis was performed according to the retention times of authentic standards. Quantitative measurement was conducted by comparing the detection results to standard curves that had been constructed according to concentrations and peak areas of reference standards in the chromatograms.

Mass spectrometry detection was performed according to the method of Wang et al. (6) with some modifications. LC-MS analysis was performed on an Agilent 1290II-G6546A UPLC-ESI-QToF mass spectrometer (Agilent Technologies, Santa Clara, CA, USA). Samples and standard alkaloids were detected by positive ionization mode with a capillary voltage of 3,000 V and a fragment or voltage of 170 V. The mass spectra were recorded from *m*/*z* 100 to 1,000. Then, the mass spectrum of individual peaks in the total ion chromatography of the extracting solution of *S. alopecuroides* seeds was compared with the mass spectrum of authentic standards of OMA, MA, OSC, SC, and SR to verify the existence of these alkaloids.

The reference standards required for the determination of bioactive compounds in *S. alopecuroides*, including OMA, MA, SR, SC, and OSC, were purchased from the National Institutes for Food and Drug Control. The chemical structures are shown in Fig. 1B.

### Total DNA extraction and high-throughput sequencing of *S. alopecuroides*

A total of 0.05 g of the sample was weighed and ground to a powder under liquid nitrogen. The total DNA of the samples was extracted using a Fast DNA SPIN kit (MP Biomedicals, Santa Ana, CA, USA), and the quality of DNA was detected by 1% agarose gel electrophoresis, while the concentration and purity of DNA were determined using NanoDrop 2000. The fungal internal transcribed spacer (ITS rDNA) was amplified using ITS1F (5′-CTTGGTCATTTAGAGGAAGTAA-3′) and ITS2R (5′-GCTGCGTTCTTCATCGATGC-3′) (29). Amplification, purification, and sequencing library construction were consistent with previous reports (2). Sequencing was performed using Illumina’s Miseq PE300 platform (Illumina, San Diego, USA) according to the standard protocols by Majorbio Bio-Pharm Technology Co., Ltd. (Shanghai, China). The raw reads were deposited into the Sequence Read Archive (SRA) database in NCBI (https://submit.ncbi.nlm.nih.gov) (SRA accession number: PRJNA832900).

### Bioinformatics analysis

The paired-end reads from sequencing were assigned to respective samples using Cutadapt software (30), based on their specificity. Barcode and primer sequences were truncated from these reads. Flash software version 1.2.7 (31) was employed to cut and splice the remaining paired-end reads of each sample, and these reads were assembled to obtain the original label. High-quality clean data were obtained by filtering the original data under specific filtering conditions (32, 33). Additionally, to segregate chimeric sequences and to eliminate the non-microbial reads, for instance, chloroplast and mitochondrial reads, the reads were compared with the Unite database (https://unite.ut.ee/) using UCHIME (34). Thus, clean reads were generated. UPARSE software version 7.0.1001 (http://drive5.com/uparse/) (35) was used to cluster the sequences into the same operational taxonomic units (OTUs) with ≥97% similarity, and a representative sequence with the highest frequency was selected for further annotation. The Unite database (Release 8.0) was used to execute annotated information for each representative sequence (36). OTUs abundance was normalized using the samples containing the least number of sequences. Subsequent analyses were based on the normalized data.

In this study, the alpha diversity indexes, including Shannoneven (representing evenness), Simpson (representing community diversity), Chao (representing community richness), and PD (representing the pedigree diversity of species), were calculated by Mothur software version v.1.30.2 (https://www.mothur.org/wiki/Download_mothur). Beta diversity distance matrix based on Bray-Curtis distance and weighted UniFrac distance was computed with QIIME. The clustering trees, Venn diagrams, and community heatmaps were generated using R software version 3.3.1. Circos plots were constructed using Circos software version 0.67-7 (http://circos.ca/).

### Co-occurrence network analysis and definition methods of the core microbiome

Co-occurrence network analysis based on Spearman correlation was used to demonstrate the relationships between different species in several samples. Endophytic fungi that were highly correlated (*r* > 0.6 or *r* < −0.6; *P* < 0.05) among all *S. alopecuroides* samples were screened to construct co-occurrence networks, which were visualized by Gephi software version 0.9.7 (https://gephi.org/). The network-level topological features [Average degree (avgK), average clustering coefficient (avgCC), average path distance (avgCC), and modularity (GD)] and the node-level topological features (degree centrality, betweenness centrality, and closeness centrality) of the *S. alopecuroides* endophytic fungal co-occurrence network were calculated in the Gephi software. In this study, the core microbiome was defined by reference to Dong et al. (37), combining membership and network connection. The genera that are common to all samples and have a relative abundance of the first 10 are defined as core microbiomes. In addition, genera whose nodes in the network met both degree centrality > 0.2, closeness centrality > 0.35, and betweenness centrality > 0.35 were defined as core microbiome (i.e., hub microbiome).

### Statistical and correlation analysis

SPSS 19.0 software (IBM Inc., Armonk, USA) was used to perform one-way ANOVA and Tukey’s test to evaluate the differences in bioactive compounds of *S. alopecuroides* at different developmental stages and organs. For indicators that did not conform to a normal distribution, the ln function was used for normal transformation. Correlations between endophytic fungal abundance and bioactive compound accumulation in *S. alopecuroides* were evaluated by Spearman analysis.

## RESULTS

### Determination of the content of medicinal compounds of *S. alopecuroides*

The HPLC detection results of the bioactive compounds of the standards and samples are shown in Fig. S1A and B. It showed that the five QAs in *S. alopecuroides* can be effectively detected under our HPLC condition. Due to the very close retention time, the SC in the analysis results represented the total amount of SC and SR. The individual mass spectrum verified the existence of these bioactive compounds (Fig. S1C through L).

**Fig 2:**
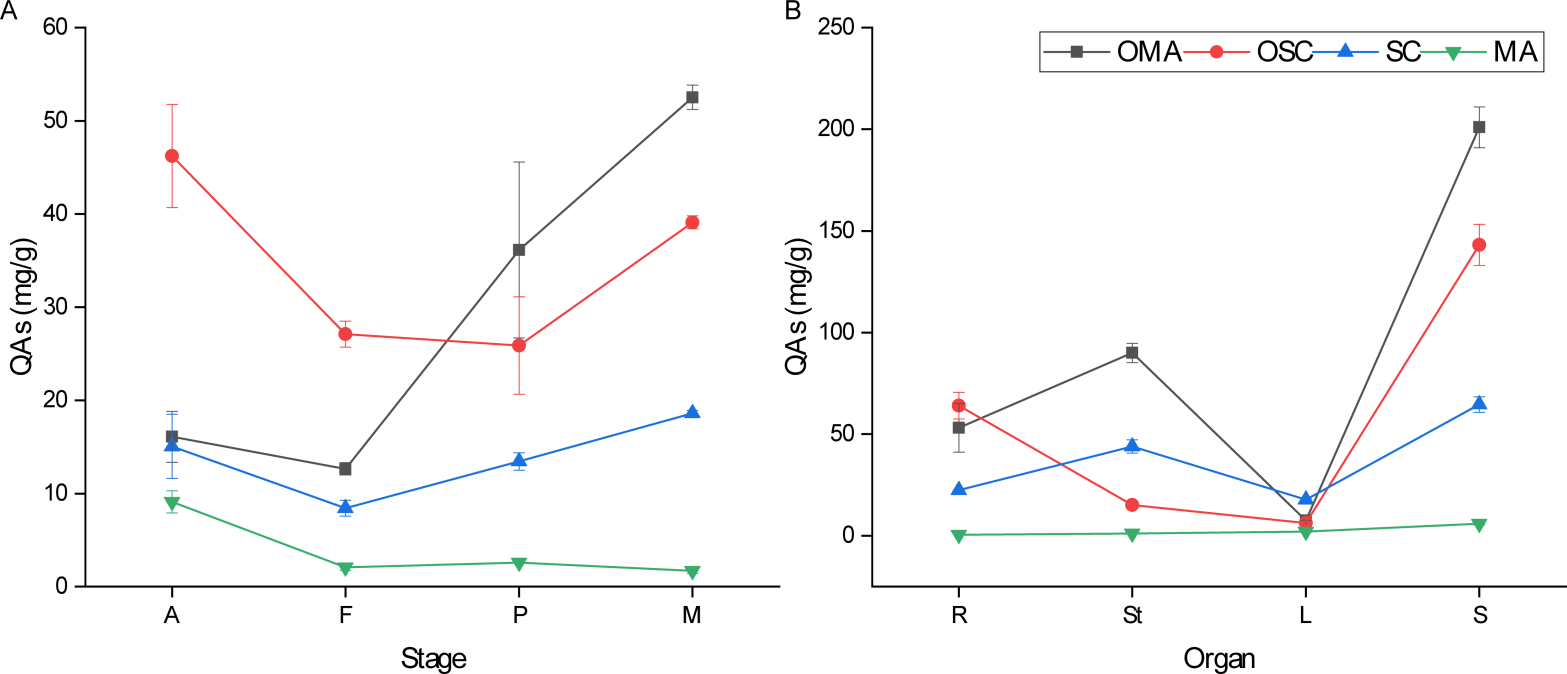
Content of quinolizidine alkaloids (mg/g) in different developmental stages (**A**) and different organs (**B**) of *S. alopecuroides*. The A, F, P, M, R, St, L, and S represent the adult stage, flowering stage, podding stage, mature stage, roots, stems, leaves, and seeds, respectively. Error bars represent mean ± SE, *n* = 3. The difference between groups was significant at the 0.05 probability level according to the ANOVA test–Tukey’s test.

The results showed that there was a significant difference (*P* < 0.05) in the content of medicinal compounds of *S. alopecuroides* at different developmental stages (Fig. 2A; Table S2). In terms of total QAs, the content of medicinal compounds in *S. alopecuroides* at the mature stage was observed to be the highest among the four developmental stages at 111.9 mg/g. This value was found to be significantly higher than the concentration observed in the flowering stage (50.2 mg/g). The highest OMA content in mature stage *S. alopecuroides* was 52.5 mg/g, which was 4.2 times higher than the OMA content in the flowering stage (12.6 mg/g). The OSC content of *S. alopecuroides* at the adult stage was 46.2 mg/g, which was 1.8 times higher than that at the podding stage (25.9 mg/g). The SC content in mature stage *S. alopecuroides* was 18.6 mg/g, which was 2.2 times higher than that in the flowering stage (8.4 mg/g). The MA content of *S. alopecuroides* at the adult stage was 9.1 mg/g, which was 5.6 times higher than that at the mature stage (1.7 mg/g).

There is a statistically significant difference (*P* < 0.05) for medicinal compounds between different organs of *S. alopecuroides* (Fig. 2B; Table S2). In terms of total QAs, the seeds contained the highest content of medicinal compounds among the four organs. The OMA content in the examined samples followed the order of seeds > stems > roots > leaves. Specifically, the OMA content in seeds was determined to be 200.9 mg/g, exhibiting a significant increase of 26.9 times compared to the OMA content in leaves (7.5 mg/g). The OSC content in the examined samples followed the order of seeds > roots > stems > leaves, with the OSC content in seeds being 143.1 mg/g, which was 22.7 times higher than the OSC content in leaves (6.3 mg/g). The SC content in the examined samples followed the order of seeds > stems > roots > leaves, with the SC content in seeds being 64.6 mg/g, which was 3.9 times higher than that in leaves (17.8 mg/g). The MA content in the examined samples followed the order of seeds > leaves > stems > roots, with the MA content in seeds being 5.9 mg/g, which was 10.3 times higher than that in roots (0.8 mg/g). This indicated that *S. alopecuroides* has the highest medicinal compound contents in the seeds, while the leaves contain the least of medicinal compounds.

### Diversity of endophytic fungi in *S. alopecuroides*

**Fig 3:**
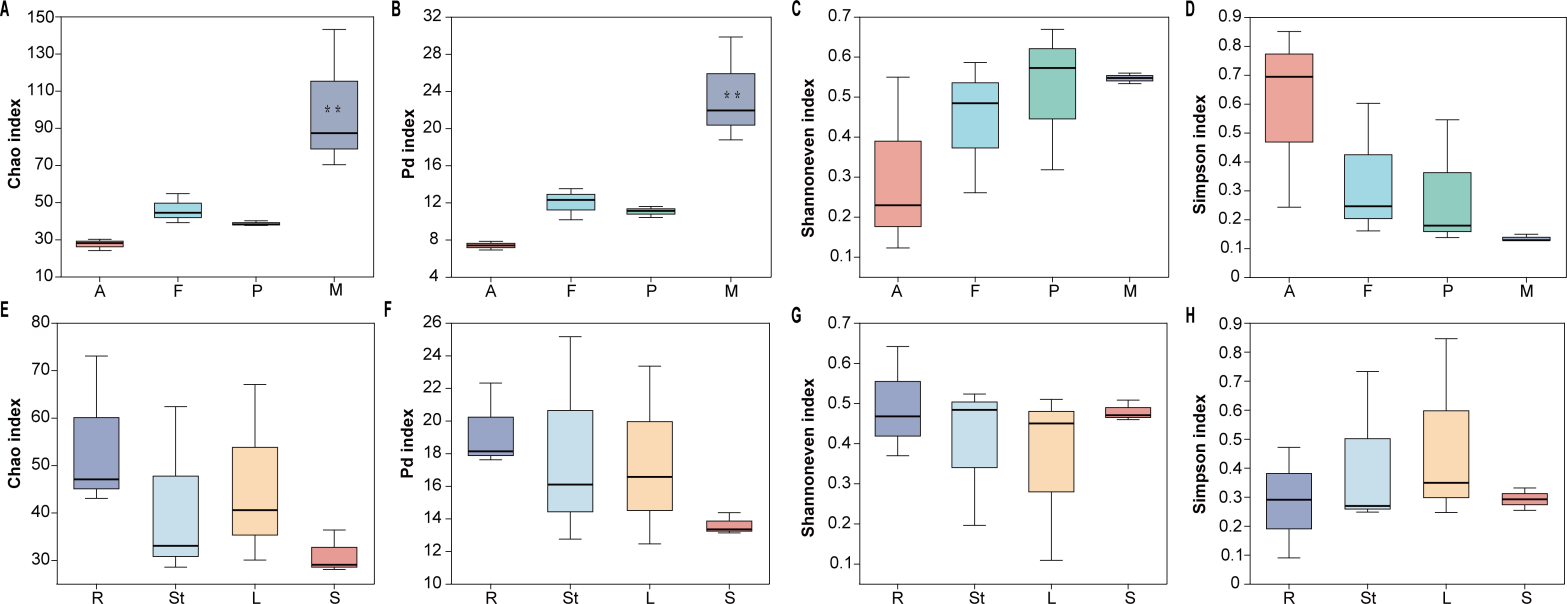
α-diversity indices of endophytic fungal communities of *S. alopecuroides* at different developmental stages (A–D) and organs (E–H). (A and E) Chao index of community richness, (B and F) PD index of species pedigree diversity, (C and G) Shannoneven index of evenness, and (D and H) Simpson index of community diversity. The letters A, F, P, M, R, St, L, and S under each boxplot represent the adult stage, flowering stage, podding stage, mature stage, roots, stems, leaves, and seeds, respectively. Error bars represent mean ± SE, *n* = 3. **P ≤ 0.01 (according to the ANOVA test–Tukey’s test).

A total of 1,227,778 high-quality sequences were obtained from high-throughput sequencing of endophytic fungi from four developmental stages of *S. alopecuroides*. The number of reads from each sample was normalized to 39,814. Finally, 477,768 effective reads were obtained and grouped into 563 OTUs based on a 97% sequence similarity threshold. The α-diversity of endophytic fungi of *S. alopecuroides* at different developmental stages was assessed by the number of sample sequences, community diversity (Simpson index), evenness (Shannoneven index), richness (Chao index), and pedigree diversity of species (PD index). The endophytic fungal community Chao index and PD index of *S. alopecuroides* at the mature stage were 100.1 and 23.5, respectively, which were significantly higher than the other three stages (*P* < 0.01). There was no significant difference in the Shannoneven and Simpson indices of endophytic fungi of *S. alopecuroides* at different developmental stages (Fig. 3). It is shown that the endophytic fungal community diversity at the mature stage was the richest and most homogeneous, and there was the greatest genetic variation during *S. alopecuroides’* growth and development.

A total of 1,464,911 high-quality sequences were obtained from high-throughput sequencing of endophytic fungi from four organs of *S. alopecuroides*. The number of reads from each sample was normalized to 37,821. Finally, 453,852 effective reads were obtained and grouped into 494 OTUs based on a 97% sequence similarity threshold. As shown in Fig. 3, the Chao index of *S. alopecuroides* endophytic fungal community showed root > leaf > stem > seed, PD index showed root > stem > leaf > seed, Shannoneven index showed root > seed > stem > leaf, and Simpson index showed leaf > stem > seed > root. Among the four organs, the root endophytic fungi had the highest Chao index, PD index, and Shannoneven index of 54.3, 19.3, and 0.4, respectively, but the lowest Simpson index of 0.3. The leaf endophytic fungi had the highest Simpson index of 0.5 and the lowest Shannoneven index of 0.4 among the four organs. Seed endophytic fungi had the lowest chao index (31.1) and PD index (13.6) among the four organs. It indicates that among the four organs of *S. alopecuroides*, the endophytic fungal community of roots was the most diverse and homogeneous with the greatest genetic differences, the endophytic fungal community of leaves was the least diverse, and the endophytic fungal community of seeds was more homogeneous than those of other organs with the smallest genetic variation.

**Fig 4:**
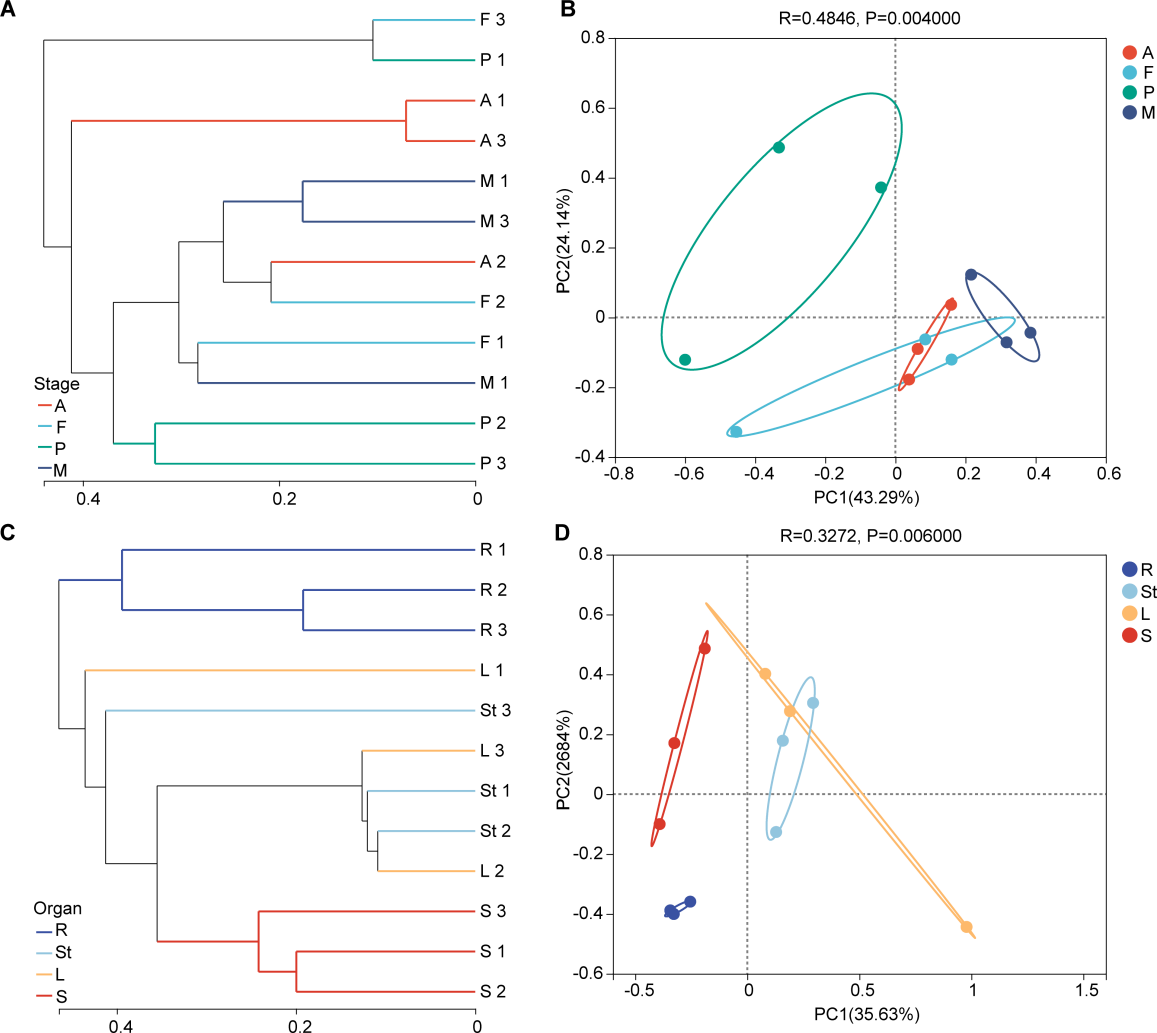
Hierarchical clustering of *S. alopecuroides* endophytic fungi based on Bray-Curtis distance (**A and C**) and PCoA analysis based on weighted UniFrac distance (different colors indicate grouped ellipses) (**B and D**). (A and B) Developmental stages and (C and D) organs in the mature stage. The A, F, P, M, R, St, L, and S represent the adult stage, flowering stage, podding stage, mature stage, roots, stems, leaves, and seeds, respectively.

Hierarchical clustering analysis with the Bray-Curtis dissimilarity algebra was applied to show the species richness information of endophytic fungi during the growth and development of *S. alopecuroides* (Fig. 4A). The endophytic fungal community richness in the four stages of *S. alopecuroides* had some differences, but the differences were small. Principal coordinates analysis (PCoA) with weighted UniFrac distance showed the diversity and evolutionary distance of endophytic fungal communities during the growth and development of *S. alopecuroides* (*R* = 0.4846, *P* = 0.0040), with 43.29% explained by the PC1 axis and 24.14% explained by the PC2 axis (Fig. 4B). Among all stages, the diversity and evolutionary distance of the endophytic fungal community of *S. alopecuroides* at the podding stage were far from the other three periods.

Hierarchical clustering analysis based on the Bray-Curtis dissimilarity algebra was applied to show the species richness information of endophytic fungi in four organs of *S. alopecuroides* (Fig. 4C). The endophytic fungal communities of *S. alopecuroides* roots showed a significant difference in abundance from those of seeds, and the abundance of stem and leaf endophytic fungal communities was in the middle of those in roots and seeds. PCoA analysis based on weighted UniFrac distances showed the richness, diversity, and evolutionary distance of endophytic fungal communities in each organ of *S. alopecuroides* (*R* = 0.3272, *P* = 0.0060), with 35.63% explained by the PC1 axis and 26.84% explained by the PC2 axis (Fig. 4D). The abundance, diversity, and evolutionary distance of endophytic fungal communities of *S. alopecuroides*’ stem and leaves were close, but those of roots and seeds were further away from each other.

### Composition of endophytic fungal community in *S. alopecuroides*

**Fig 5:**
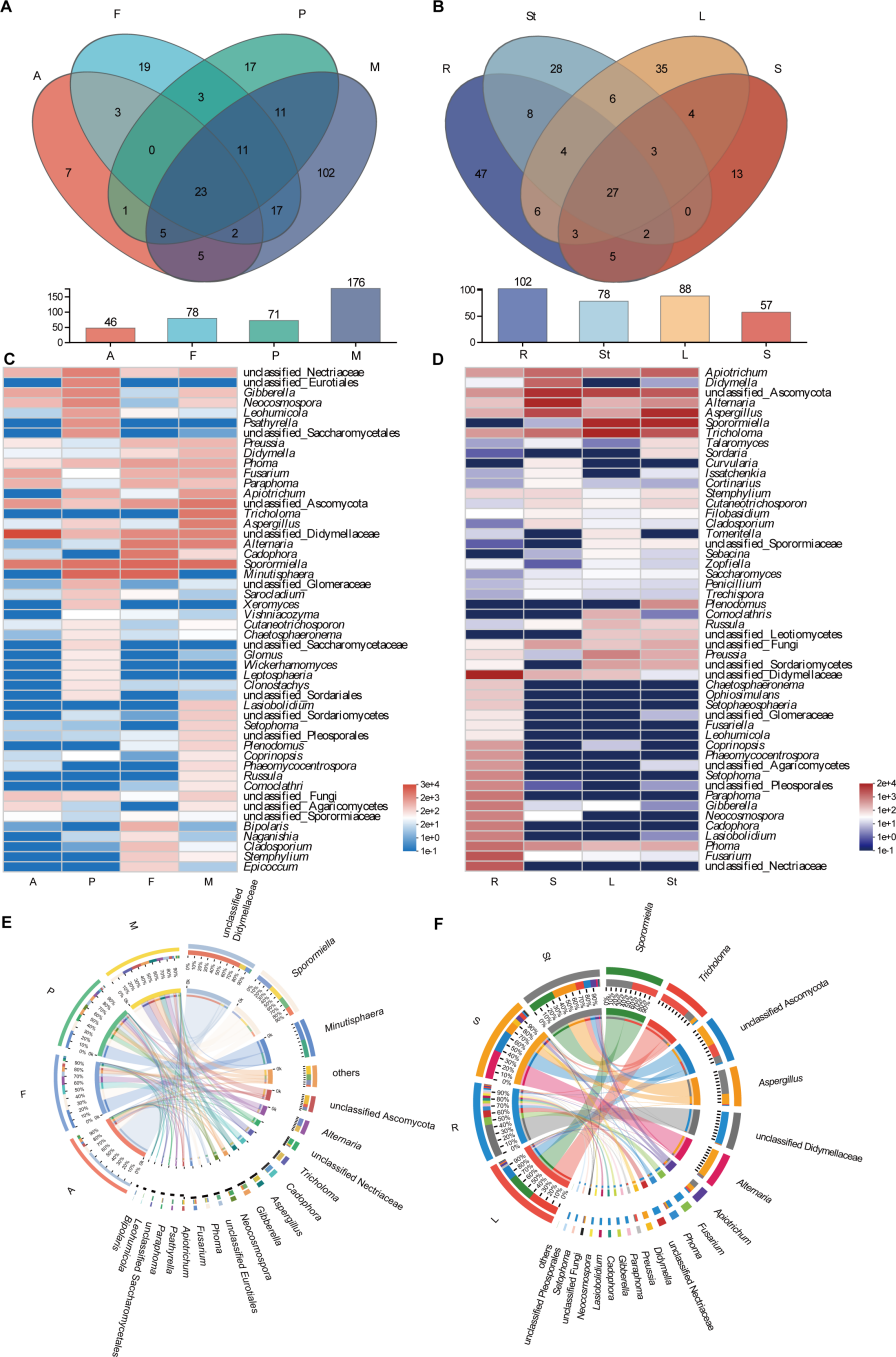
Composition of endophytic fungal community in *S. alopecuroides*. (A and B) Venn diagram, (C and D) heatmap of first 50 genera, (E and F) Circos diagram of first 20 genera. (A, C, and E) Developmental stages and (B, D, and F) organs in the mature stage. The A, F, P, M, R, St, L, and S represent the adult stage, flowering stage, podding stage, mature stage, roots, stems, leaves, and seeds, respectively.

The endophytic fungal community composition of the four developmental stages of *S. alopecuroides* was divided into 9 phyla, 31 classes, 68 orders, 133 families, and 226 genera. The Venn diagram showed the number of individuals and shared genera in the endophytic fungal community of *S. alopecuroides* (Fig. 5A). *S. alopecuroides* had the most endophytic fungal species in the mature stage, followed by the flowering stage, then the podding stage, and finally, the adult stage. The largest number of endophytic fungal species, with 102 genera, was unique to the mature stage, while the least number of endophytic fungi was unique to the adult stage with seven genera. There were 23 shared genera of endophytic fungi in different developmental stages of *S. alopecuroides* (Fig. 5A), among which the first five genera in terms of richness were unclassified Didymellaceae, *Sporormiella*, unclassified Ascomycota, *Alternaria,* and unclassified Nectriaceae, and they were accounted for 31.2%, 24.3%, 8.1%, 7.3%, and 5.0% of the total shared genera, respectively (Fig. S2A).

The endophytic fungal community composition in different tissues and organs of *S. alopecuroides* was divided into 7 phyla, 25 orders, 58 classes, 115 families, and 191 genera. The most abundant species of endophytic fungi were found in roots, followed by leaves, then stems, and finally seeds. The largest number of endophytic fungi species in roots was 47 genera, followed by leaves and stems with 35 and 38 genera, respectively, and the smallest number of specific endophytic fungi species was in seeds with 13 genera. There were 27 shared genera of endophytic fungi from different organs of *S. alopecuroides* (Fig. 5B), among which the top five by richness were *Tricholoma*, unclassified Ascomycota, *Aspergillus*, unclassified Didymellaceae, and *Alternaria*, which were accounted for 18.4%, 18.0%, 15.4%, 14.3%, and 13.4% of the total shared genera, respectively (Fig. S2B).

To gain insight into the dynamic of diversity and population for endophytic fungi during different developmental stages of *S. alopecuroides*, the relative abundance at the level of the first 50 endophytic fungal genera heatmap (Fig. 5C) and the relationship map (Fig. 5E) of the first 20 genera were plotted. The dominant endophytic fungi at the adult stage were the unclassified Didymellaceae with a relative abundance of 70.8%, followed by *Sporormiella* with a relative abundance of 11.4%. The dominant endophytic fungi at both the flowering and the podding stages were *Minutisphaera* with relative abundance of 25.6% and 24.3%, followed by *Sporormiella* (22.2% and 15.3%, respectively). The dominant endophytic fungi at the mature stage were *Sporormiella* (19.4%), followed by *Tricholoma* (13.6%).

The heatmap of the first 50 genera for the richness of the four organs of the *S. alopecuroides* (Fig. 5D) and the relationship map of the first 20 genera (Fig. 5F) showed that unclassified Didymellaceae had the highest relative richness in the roots with 40.6%. *Sporormiella* had the highest relative abundance in stems with 32.6%, followed by *Aspergillus* with 29.8%. *Tricholoma* had the highest relative abundance in the leaves with 32.6%. *Alternaria* and unclassified Ascomycota had the highest relative abundance in the seeds with 35.8% and 26.6%, respectively. Based on the result, we defined the first 10 relative abundance genera common to all samples to be the core microbiome (bolded in Fig. S2).

### Co-occurrence network analysis of endophytic fungi in *S. alopecuroides*

**Fig 6:**
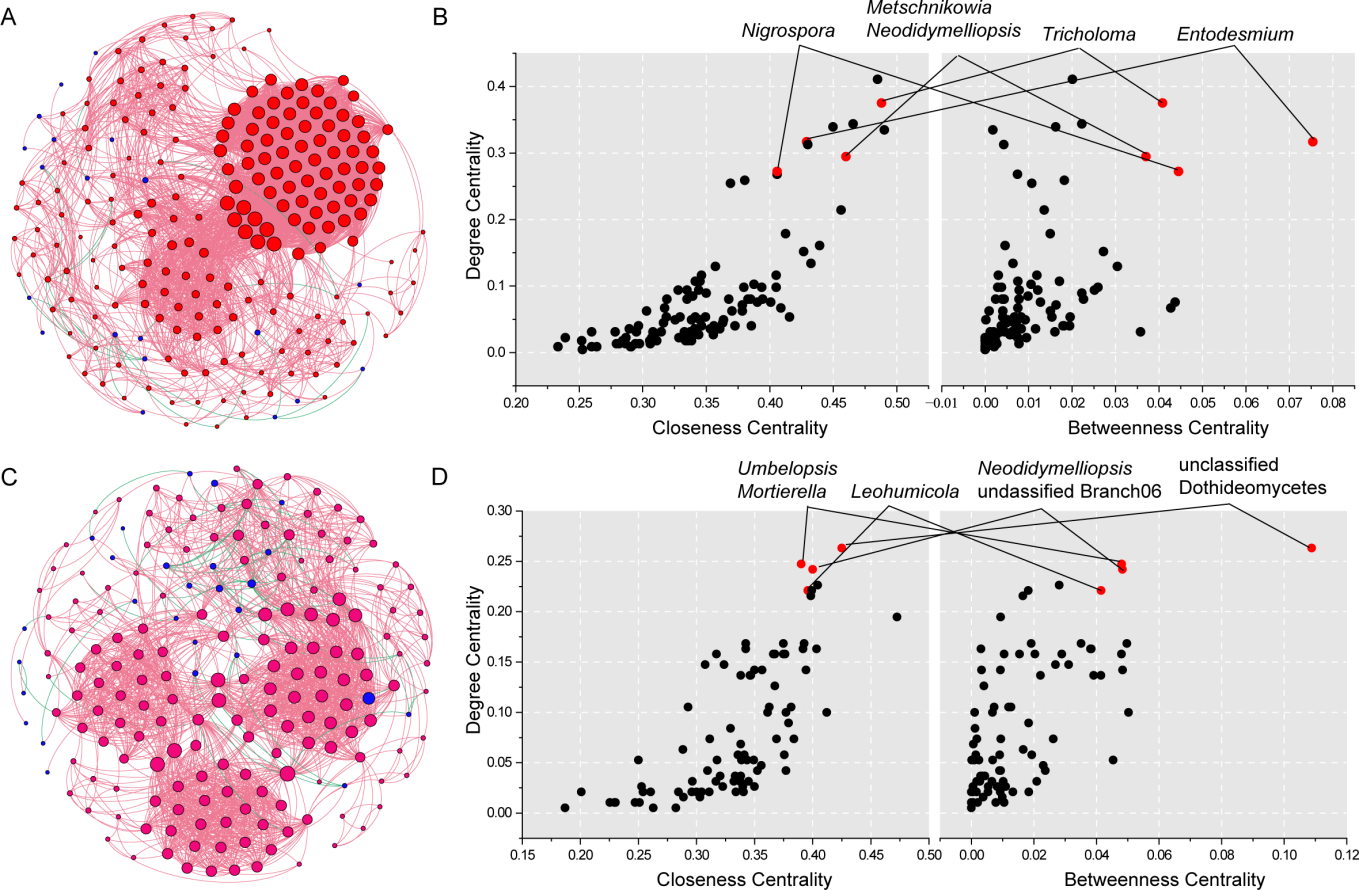
Co-occurrence network analysis of endophytic fungi in *S. alopecuroides*. (A and B) Developmental stages and (C and D) organs at the mature stage. (A and C) Correlation co-occurrence network of endophytic fungi from *S. alopecuroides*. The nodes represent the genera. Node size has been mapped to reflect the degree centrality. Blue nodes indicate the core microbiome. Red edges represent positive correlations (*R* > 0.6 and *P* < 0.05) and green edges represent negative correlations (*R* < −0.6 and *P* < 0.05) according to Spearman’s correlation. Line thickness represents the size of the correlation. (B and D) “Core microbiomes” were defined as those genera that were significantly more central (measures of node importance) based on the degree centrality > 0.2, betweenness centrality >0.35, and closeness centrality > 0.35.

There were differences in the interaction patterns between the two endophytic fungi of *S. alopecuroides*. As shown in Fig. 6A, the *S. alopecuroides*’ endophytic fungal co-occurrence network at developmental stages contained 225 nodes and 4,065 edges. Among those nodes and edges, 99.5% are positively correlated edges and 0.5% are negatively correlated edges. The analysis of the network topology revealed that the co-occurrence network exhibited an average connectivity (avgK) of 36.1, an average clustering coefficient (avgCC) of 0.7, an average path distance (GD) of 2.6, and a modularity value of 0.4. The *S. alopecuroides’* organ endophytic fungal co-occurrence network consisted of 191 nodes and 1,970 edges (Fig. 6C). Among those nodes and edges, there are 96.6% positively correlated edges and 0.5% negatively correlated edges. The organ’s co-occurrence network exhibited an average connectivity (avgK) of 20.6, an average clustering coefficient (avgCC) of 0.7, an average path distance (GD) of 2.9, and a modularity value of 0.6. These findings suggest that the endophytic fungal interactions’ network of *S. alopecuroides* during its developmental stages is characterized by a relatively complex structure, with a higher degree of modularity and tightly interconnected nodes within the module.

In this study, the core microbiomes were identified based on three network topological characteristics (degree centrality > 0.2, closeness centrality > 0.35, and betweenness centrality > 0.35) (Fig. 6B and D). Five core endophytic fungi, *Tricholoma*, *Metschnikowia*, *Neodidymelliopsis*, *Nigrospora*, and *Entodesmium*, were found in the mutualistic network of endophytic fungi of *S. alopecuroides* at the developmental stages. Six core endophytic fungi were found in the intercrossing network of endophytic fungi in the mature stage of *S. alopecuroides*, namely *Leohumicola*, unclassified Branch06, *Neodidymelliopsis*, unclassified Dothideomycetes, *Umbelopsis*, and *Mortierella*.

### Correlation analysis of the core microbiomes in *S. alopecuroides* and their medicinal compounds

**Fig 7:**
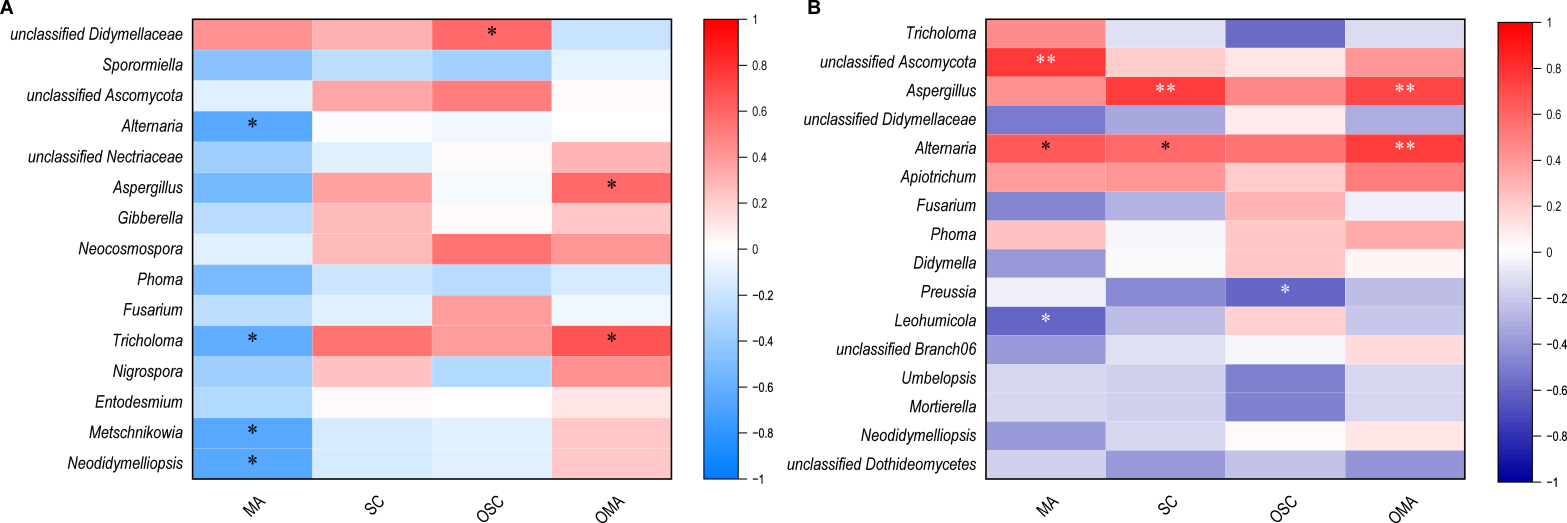
Spearman correlation analysis of core microbiomes in *S. alopecuroides* and its medicinal compounds at different developmental stages (**A**) and organs (**B**). *P < 0.05 and **P < 0.01.

Determined by the combined membership and network connection, there were 15 endophytic core microbiomes at different developmental stages and 16 endophytic core microbiomes at different organs in *S. alopecuroides* at the mature stage. The relationship between the core microbiome and the content of medicinal compounds in different organs in the reproductive period and at the mature stage was evaluated by heatmaps of Spearman’s correlation coefficient (Fig. 7). Unclassified Didymellaceae (0.6) at different developmental stages were significantly positively correlated to OSC (*P* < 0.05). *Aspergillus* (0.6) and *Tricholoma* (0.7) were significantly positively correlated with OMA (*P* < 0.05), while *Alternaria* (−0.7), *Tricholoma* (−0.6), *Metschnikowia* (−0.7), and *Neodidymelliopsis* (−0.7) were significantly negatively correlated with MA (*P* < 0.05) (Fig. 7A). At the mature stage, unclassified Ascomycota (0.8) from different organs was highly significantly positively correlated with MA (*P* < 0.01). *Aspergillus* showed a highly significant positive correlation (*P* < 0.01) with SC (0.8) and OMA (0.7). *Alternaria* was significantly positively correlated (*P* < 0.05) with MA (0.6) and SC (0.6) and highly significantly positively correlated (*P* < 0.01) with OMA (0.8). While *Leohumicola* and *Preussia* were significantly negatively correlated (*P* < 0.05) with MA (0.6) and OSC (0.6), respectively (Fig. 7B).

## DISCUSSION

In traditional Chinese herb medicine, the whole plant of *S. alopecuroides* is used as a medicinal botanical product in clinics. It is an important resource for the industrial extraction of QAs, which are used in the pharmaceutical manufactories (6). Our results showed that the alkaloid content varies significantly with developmental stages and organs, and it is consistent with the results of the previous study by Lin et al. on the effect of seasonal changes on the alkaloid yield of *Festuca sinensis* (38). Furthermore, we identified that the total alkaloid content of the plant is at its highest in the mature stage, which is identical with the compounds’ analytic results of fall harvesting *S. alopecuroides* (Fig. 2; Table S2). This study also found that OMA and OSC are the two most abundant alkaloids in different developmental stages and organs (Table S2), which is also consistent with the findings of Wang et al. (6).

Previous research has shown that endophytic fungi can be found in all growth stages and tissues of a plant (16), with the diversity and abundance of endophytic fungi varying in different developmental stages and organ tissues (39). Of *S. alopecuroides*, our results showed that the endophytic fungal community can dynamically change in different developmental stages (Fig. 3). Endophytic fungi can be transmitted both horizontally (non-systematic transmission) and vertically (systematic transmission). At different stages of plant growth and development, endophytic fungal communities are sensitive to horizontal fungal transmission (40). Numerous studies have shown that plant endophytic fungi have different host preferences and tissue specificities (41). For example, endophytic fungal communities in roots are more diverse than in other organs (42). Consistent with the other research, in this study, the highest abundance and diversity of endophytic fungi were found in the roots of *S. alopecuroides*, followed by stems, leaves, and seeds. The greatest evolutionary distance of endophytic fungal communities was found between the roots and seeds (Fig. 3 and 4). This result can be explained by the following factors: (i) Roots secrete exudates that attract beneficial microorganisms from the soil. These microorganisms can enter the roots through dead roots and lenticels (42, 43). (ii) Microorganisms must colonize the plant leaf surface first and then enter the interior of the leaf tissue through stomata, water pores, or wounds (44). (iii) Seeds are relatively isolated from the environment, and it is difficult for external microorganisms to enter the seeds (45, 46).

The membership definition method is the most common method for defining the core microbiome (37). It identifies the common taxa in the host microbiome by comparing whether there are shared OTUs (37). These taxa have been vertically transmitted, recruited, selected, and inherited through evolution (37). They are also highly competitive with other microorganisms and are mostly potentially functional microorganisms closely related to plants (47, 48). Numerous studies have concluded that vertically transmitted endophytic fungi are mutually beneficial to their hosts and are the result of long-term selective evolution with the host plant (40). During seed maturation, the relative abundance of endophytes changes significantly, and vertically transmitted endophytes become the predominant endophyte species of the seed microbial community (45). Our result shows that the common endophytic fungi existing in all developmental stages of *S. alopecuroides,* such as unclassified Ascomycota, *Aspergillus*, and *Alternaria*, were most likely to be vertically transmitted. Although they did not show dominance in the reproduction period, the vertically transmitted endophytic fungi showed statistically significant (*P* < 0.05) or highly significant (*P* < 0.01) positive correlations with host MA, SC, and OMA at seed maturation (Fig. 5 and 7; Fig. S2). The result indicates that many vertically transmitted endophytic fungi are retained in the seeds when *S. alopecuroides* grow to the mature stage. With the development of the plant, these endophytes gradually show dominance, resulting in the occupation of favorable ecological niches, and further influencing host metabolism. This result was also supported by numerous studies of other plants. Previous research using the membership definition method to determine the core endophytic fungi of *Salvia miltiorrhiza* showed that the biosynthesis of terpenoid, limonene degradation, pinene, geraniol, and prenyltransferases wasoverrepresented in the core microbiome (48). Harrison et al. (49, 50) also showed that the vertically transmitted endophytic fungus *Alternaria fulva* (belonging to *Alternaria* sect. *Undifilum*) can affect the leaf fungal endophyte microorganisms of *Astragalus* and directly influence the plant traits.

Microbial communities are not independent entities. Instead, they form complex interactions in each individual species through symbiosis or competition (42, 51). These interactions further form a complex mutual biological network, which sustains the homeostasis, structure, and function of the biological system in the plant (10). The composition of microbial communities and their symbiotic networks can dramatically influence the biological function of the host plant (24, 52, 53). From the network, the core microorganisms (hub taxa) can be identified in the microbial community by the co-occurrence network. Hub taxa affect the community structure through stronger biological interactions with the host or other microbial species. It is important to note that, besides focusing on the high numbers of abundance and composition, we should also focus on the ecological significance of hub taxa in the complex biological system (37, 54). Hub taxa not only represent key species that have strong direct and indirect effects on the microbiome but also play an intermediary role between plants and their associated microbiomes (52). A typical example is the core microbiome (i.e., hub microbes) defined as *Albugo* and *Dioszegia* based on network parameters, degree centrality, closeness centrality, and betweenness centrality in *Arabidopsis*. These core microbiomes play a crucial role in the development of the *Arabidopsis* leaf layer microbiome and the maintenance of plant health (54). In this study, we used the nodal attributes of the symbiotic network to define the core microbiome. These microorganisms identified as core microbiome played a complementary role to the core microbiome defined by the membership definition method, although they are not present in all samples. Furthermore, we also found that the endophytic fungi defined by the co-occurrence network had no relationship with *S. alopecuroides*’ QAs, and their ecological functions need to be further investigated.

Endophytic fungi play a major role as endogenous environmental factors affecting the quality of medicinal herbs in pharmaceutical manufacturing (10). The relationship between endophytic fungi and their host active compounds is a hot topic of the research area. Several studies have reported that endophytic fungi of medicinal plants can affect the amount of host medicinal compounds, such as *Salvia miltiorrhiza* (48), *Huperzia serrata* (55), *licorice* (56), and *Astragalus* and *Oxytropis* (57). These studies show that the presence of fungi is significantly associated with the biosynthesis of medicinal compounds in medicinal plants (48, 55–57). In a previous study, the endophytic fungi of *Alternaria* showed a significant positive correlation between endophytic fungi and QAs in *S. alopecuroides* across different geographical areas (2). Notably, in this study, endophytic *Alternaria* of different organs of *S. alopecuroides* at the mature stage showed a highly significant (*P* < 0.01) positive correlation with QAs (Fig. 7), where QAs are strong quality control for the medicinal properties of *S. alopecuroides*. The non-significant correlation between *S. alopecuroides*’ endophytic fungi and QAs in different developmental stages could be explained by the dilution effect of horizontally transmitted endophytic fungi on vertically transmitted fungi before the seed mature stage. Together with the influence of the external environment on the plant, the influence of endophytic fungi was not significantly evident. While at the mature stage, vertically transmitted endophytic fungi gradually dominate the entire community (45).

This study identified *Alternaria* as a key endophytic fungus that affects the medicinal composition of *S. alopecuroides*. Other studies have shown that *Alternaria oxytropis* forms a symbiotic relationship with *Oxytropis ochrocephala. Alternaria oxytropis* is the key strain that produces swainsonine in *Oxytropis ochrocephala*, and it is also the direct producer of swainsonine (58, 59). *Alternaria* fungi are themselves a huge reservoir of secondary metabolites, producing numerous bioactive substances, such as vincaleukoblastinum (60), resveratrol (61), and some alcohols (59). Thus, our next study will focus on two aspects: further investigating the mechanism by which endophytic *Alternaria* promotes the synthesis and accumulation of medicinally bioactive compounds in the host. Identifying the metabolites of *Alternaria*, screening for endophytic fungal strains that produce QAs, and developing new methods for the development and utilization of these functional strains.

## Data Availability

The data sets presented in this study can be found in the supplemental material and under PRJNA832900.

## SUPPLEMENTAL MATERIAL

Tables S1 and S2, Figures S1 and S2 spectrum.03076-23-s0001.pdf Table S1 (Standard curve in the detection of bioactive compounds in *S. alopecuroides*), Table S2 (Content of quinolizidine alkaloids (mg/g) in different developmental stages and different organs of *S. alopecuroides*), Fig. S1 (High-performance liquid chromatograms [A and B] and the individual mass spectrum [C~L] of the bioactive compounds of the five alkaloids, standard and representative samples), and Fig. S2 (The percentage of shared genera in *S. alopecuroides*).

ASM does not own the copyrights to Supplemental Material that may be linked to, or accessed through, an article. The authors have granted ASM a non-exclusive, world-wide license to publish the Supplemental Material files. Please contact the corresponding author directly for reuse.
