# Supplementary material for: Community ecological succession of endophytic fungi associates with medicinal compound accumulation in Sophora alopecuroides
Source: Microbiol Spectr. 2024 Jan 18;12(2):e03076-23. doi: 10.1128/spectrum.03076-23 (PMC10845968; doi:10.1128/spectrum.03076-23)
Supplement: Tables S1 and S2, Figures S1 and S2 — Table S1 (Standard curve in the detection of bioactive compounds in S. alopecuroides), Table S2 (Content of quinolizidine alkaloids (mg/g) in different developmental stages and different organs of S. alopecuroides), Fig. S1 (High-performance liquid chromatograms [A and B] and the individual mass spectrum [C~L] of the bioactive compounds of the five alkaloids, standard and representative samples), and Fig. S2 (The percentage of shared genera in S. alopecuroides). [file spectrum.03076-23-s0001.pdf]

Supplementary Material

Supplementary Table 1 Standard curve in the detection of bioactive compounds in *S. alopecuroides*

compound	linear regression*	R2	detection wavelength (nm)	linear range (mg/mL)
OMA	y=1434.1x+25.131	0.9998	205	0.078~10.000
OSC	y=1812.7x+62.971	0.9993	205	0.040~5.000
SC	y=1449.7x+28.963	0.9996	205	0.200~2.500
MA	y=1240.5x+9.9532	0.9995	205	0.005~0.600

Note: *: The x value is the concentration of the analyte (mg/mL), and the y value is the peak area of the tested compound.

Supplementary Table 2 Content of quinolizidine alkaloids (mg/g) in different developmental stages and different organs of *S. alopecuroides*

Sample	Mean ± LSD (n=3)				
	OMA	OSC	SC	MA	Total QAs
A	16.1±2.7bc	46.2±5.5a	15.1±3.4ab	9.1±1.2a	86.5±12.8ab
F	12.6±0.7c	27.1±1.4b	8.4±0.9b	2.1±0.3b	50.2±1.9b
P	36.1±9.4ab	25.9±5.2b	13.4±0.9ab	2.6±0.1b	78.0±14.4ab
M	52.5±1.3a	39.1±0.7ab	18.6±0.3a	1.7±0.3b	111.9±0.0a
R	53.2±12.0B	64.1±6.6B	22.5±0.2C	0.6±0.0B	140.3±18.5B
St	89.9±4.7B	15.1±0.2C	44.0±3.2B	1.1±0.2B	150.2±5.1B
L	7.5±0.2C	6.3±1.0C	17.8±0.5C	2.0±0.2B	33.6±1.1C
S	200.9±10.0A	143.1±10.1A	64.6±3.8A	5.9±0.6A	414.5±17.8A

Note: The A, F, P, M, R, St, L, and S represent the adult stage, flowering stage, podding stage, mature stage roots, stems, leaves, and seeds, respectively. Lowercase letters (a, b, c) represent the significant difference (*P* <0.05) between developmental stages and capital letters (A, B, C) represent the significant difference (*P* <0.05) between organs according to the ANOVA test—Turkey's test.

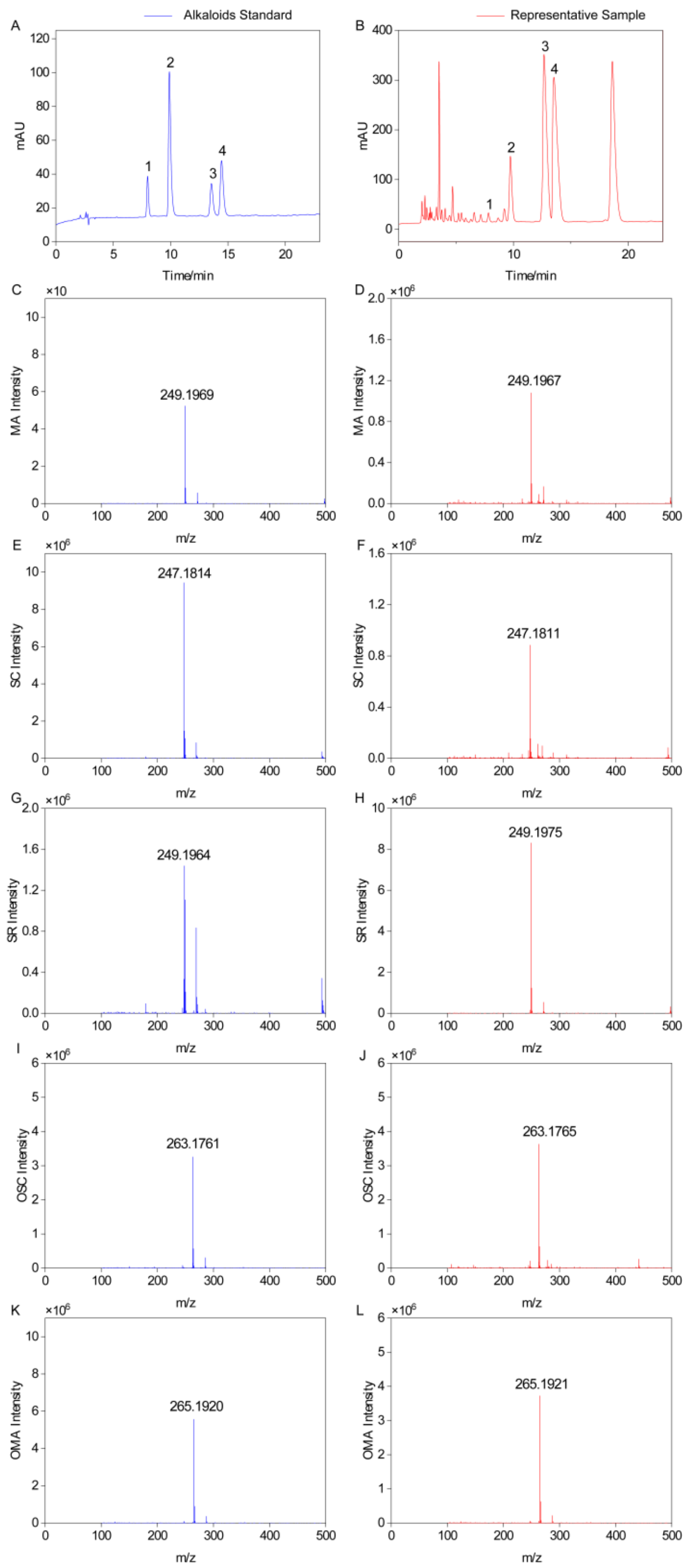

Supplementary Figures 1 High-performance liquid chromatograms (A and B) and the individual mass spectrum (C~L) of the bioactive compounds of the five alkaloids standard and representative sample. The blue and red colors represent the alkaloids' standard and representative samples, respectively. Numbers (1, 2, 3, and 4) represent matrine (MA), sophocarpine and sophoridine (SC+SR), oxysophocarpine (OSC), and oxymatrine (OMA), respectively.

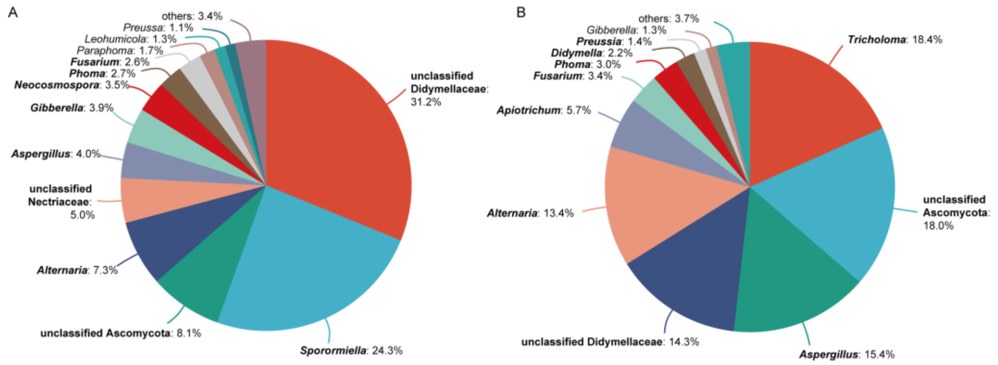

Supplementary Figures 2 The percentage of shared genera in *S. alopecuroides*. A: developmental stages, B: organs in the mature stage. The bolded genera are defined as the core microbiome.
